# Posterior Reversible Encephalopathy Syndrome Onset Within 24 Hours Following Moderna mRNA Booster COVID-19 Vaccination: Vaccine Adverse Event Vs. Hypertension?

**DOI:** 10.7759/cureus.24919

**Published:** 2022-05-11

**Authors:** Jocelyn McCullough, Manal Ahmad, Idy Tam, Reid Portnoy, Joseph Ng, Kuschner Zachary, Alan Kaell

**Affiliations:** 1 Medical Education, Mather Hospital, Commack, USA; 2 Medical Education, Zucker School of Medicine, Hempstead, USA; 3 Medicine, Zucker School Of Medicine, Hempstead, USA; 4 Medical Education, Zucker School Of Medicine, Hempstead, USA; 5 Pulmonary and Critical Care Medicine, Zucker School Of Medicine, Hempstead, USA; 6 General Medicine, Zucker School Of Medicine, Hempstead, USA; 7 Medicine, Zucker School of Medicine, Hempstead, USA

**Keywords:** hypertension and covid-19, irreversible neurological damage, posterior reversible encephalopathy syndrome (pres), covid 19 vaccine complication, primary hypertension, vaccine adverse events

## Abstract

We present a case of a female who presented with the acute onset of neurological changes within 24 hours of receiving her third, or booster, dose of the mRNA Moderna (Cambridge, Massachusetts) coronavirus disease 2019 (COVID-19) vaccination. Her clinicoradiological findings were most consistent with posterior reversible encephalopathy syndrome (PRES). Although PRES has been reported with severe acute respiratory syndrome coronavirus 2 (SARS-CoV-2) infection, this raised suspicion of a possible vaccine-induced PRES with her only confounder being hypertension managed with a beta-blocker. Extensive workup for other entities associated with PRES, including infection, autoimmune, paraneoplastic syndrome, and alcohol were unrevealing. Thus far, there have not been any reports of PRES post mRNA vaccination. We encourage providers to report similar cases with neurological manifestations post mRNA vaccination to the vaccine adverse event reporting system (VAERS). Timely diagnosis and treatment of PRES may help minimize any irreversible neurological sequelae.

## Introduction

Posterior reversible encephalopathy syndrome (PRES) is a clinicoradiological syndrome [1-5]. It usually presents with acute onset of headaches, seizures, altered mental status, visual loss, and associated white matter vasogenic edema affecting the posterior occipital and parietal lobes of the brain on imaging. The incidence of PRES is unknown but based on case series, there seems to be a predominance in women compared to men. There have been many theories associated with the pathogenesis of PRES, including hypertension, endothelial injury, side effects of certain chemo medications, and, more recently, there have been cases of coronavirus disease 2019 (COVID-19)-induced PRES [6-11]. However, there have not been any cases reported post-COVID-19 messenger RNA (mRNA) vaccination. Although PRES was thought to be reversible, there are well-documented cases of irreversible neurologic sequelae that do not always correlate with MRI improvement [11]. We present an interesting case of PRES syndrome abruptly occurring within 24 hours of the Moderna (Cambridge, Massachusetts) COVID-19 booster mRNA vaccination.

## Case presentation

A 76-year-old female presented to our ED with acute onset confusion, unsteady gait, and blurry vision within 24 hours after receiving the mRNA Moderna booster vaccine. Medical history was notable for uncomplicated hypertension on metoprolol, alcohol use disorder without complications, and recent shingles limited to dermatome L1, resolving promptly after initiation of famciclovir within 24 hours and completing the dosage two weeks prior to her Moderna booster. On physical examination, the patient was afebrile, awake, oriented intermittently x 3, with periods of confusion, blurry vision, intermittent unsteady gait, and normal speech. Her neuro exam was negative without any focal motor, sensory or cerebellar deficits, normal gait, extraocular muscles (EOM) full, with normal fundi and corrective acuity. The patient’s blood pressure was 192/80 mmHg bilaterally in both arms. Both chest X-ray and EKG were within normal limits.

 The patient’s ethyl alcohol level was <10 mg/dL, serum calcium elevated at 13.5 mg/dL (nl < 10.3 mg/dL), vitamin D 25-OH 200 ng/mL (upper limit of normal (ULN) <50 ng/ml). Parathyroid hormone was 23 pg/mL (15-65 pg/mL) and parathyroid-related hormone was <2.0 pmol/L, respectively. Angiotensin-converting enzyme (ACE) was 17 U/L (normal 4-82U/L. Renal artery stenosis was negative on ultrasound. Hypertension was managed with a home dose of metoprolol 150 mg/day, and hypercalcemia resolved with intravenous (IV) fluids. 

On the third day of admission, the patient was found lying in bed, unresponsive to sternal rub with motor twitching of the left arm consistent with seizure-like activity and was presumed to be obtunded due to a post-ictal state. She was transferred to the intensive care unit (ICU) for intubation for airway protection and treatment with diazepam, elevated blood pressure of 185/104 mmHg, and fever of 100.8 F. Empirical antibiotics with vancomycin, ampicillin, and acyclovir for presumed central nervous system (CNS) bacterial and viral encephalitis were initiated. EEG demonstrated seizure foci in bilateral posterior quadrants. The lumbar puncture showed normal opening pressure, with a normal cell count of 1 mm^3^ and glucose of 70 mg/dL (nl < 80 mg/dL) but an elevated total protein of 95.9 mg/dl (nl < 60 mg/dL), cerebrospinal fluid (CSF) fluid viral polymerase chain reaction (PCR)/culture was negative for herpes simplex virus (HSV)1/2, West Nile virus (WNV), and cytomegalovirus (CMV). Acid-fast bacilli (AFB) smear and cultures were negative.

**Figure 1:**
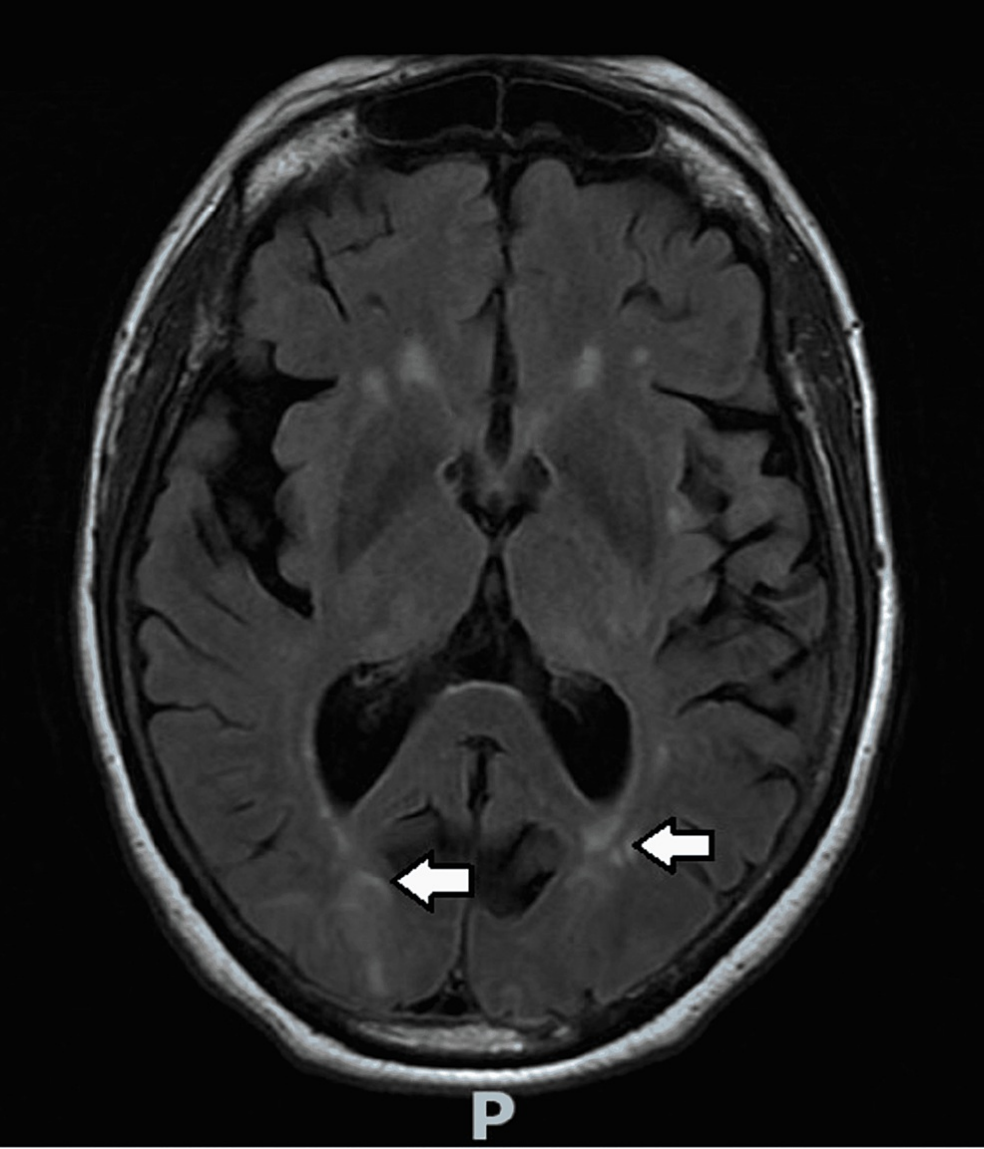
MRI of the brain without contrast The white arrows represent interval development of T2/fluid-attenuated inversion recovery (FLAIR) hyperintensities in the parieto-occipital lobes, which represent acute posterior reversible encephalopathy.

Extensive workup, including antinuclear antibody, anti-double-stranded antibody 12 IU/ml, anti-myeloperoxidase antibody <9 U/mL, anti-proteinase <3.5 U/mL, anti-cytoplasmic <1:20 titer, anti-perinuclear <1:20 titer, atypical p-ANCA <1:20; additionally, paraneoplastic neurological antibodies, including anti-Hu, anti-Ri, anti-Yo, and anti-ganglioside antibodies were all negative. Serum protein electrophoresis showed globulin 2.5%, albumin: globulin ratio 1.4, without any monoclonal band, and urine protein electrophoresis showed urine alpha-1 globulin 2.5%, alpha-2 globulin 7%, and beta globulin 16.2%, without M-spike. Immunoglobulins and flow cytometry were not suggestive of myeloproliferative disease and paraneoplastic encephalopathy workup was negative. CT of the chest, abdomen, and pelvis with contrast was negative for neoplasm. MRI imaging on Day # 3 showed T2/fluid-attenuated inversion recovery (FLAIR) hyperintensities in the parieto-occipital lobes and pulvinar of the thalami suggestive of PRES (Figure 1). Neurology thought her clinical presentation and neuroimaging were much more consistent with PRES than with acute disseminated encephalomyelitis and rarely reported post-meningococcal vaccination [12].

**Figure 2:**
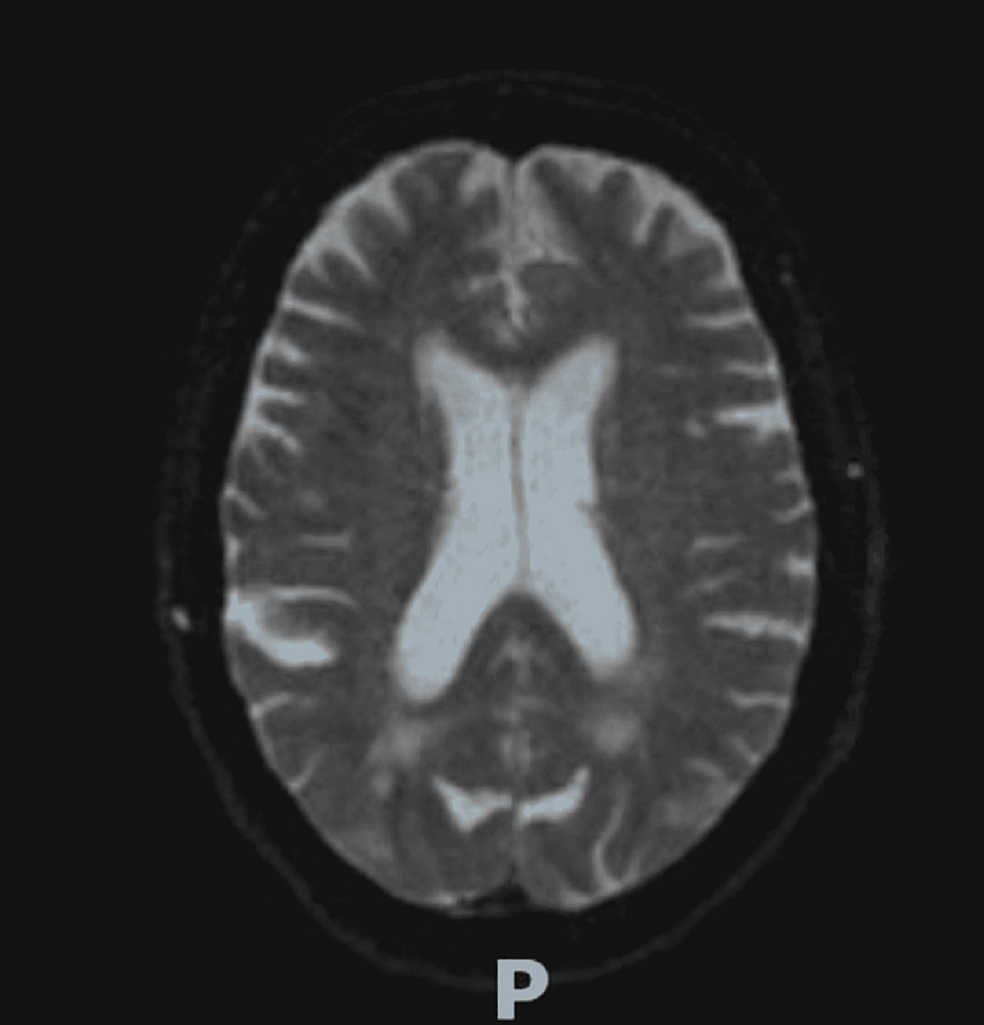
MRI with contrast showing previously seen white matter changes with PRES appear largely resolved PRES: posterior reversible encephalopathy syndrome

Her hypertension was aggressively managed with amlodipine, enalapril, and intravenous (IV) metoprolol to decrease cerebral perfusion pressure while avoiding IV nitroglycerin, known to risk lowering CNS perfusion and exacerbating PRES [13]. Empiric steroids were not given, and antiviral and antibacterial agents were discontinued after 72 hours, Levetiracetam 500 mg twice daily was utilized as an anti-seizure medication. Repeat MRI imaging on Day #14 demonstrated a large resolution of the hyperintensities without episodic blurry vision, confusion, or further seizures (Figure 2). Her mentation improved to follow two-step commands, but deconditioning, residual slow mentation, diminished gag reflex, aphasia, failed swallow evaluation, and failed breathing trials required both tracheostomy and percutaneous endoscopic gastrostomy (PEG) tube placement and transfer to an acute rehabilitation center on the 28th day of admission. She was discharged on levetiracetam 750 mg twice daily, and an anti-hypertensive regimen of nimodipine 60 mg every four hours via a PEG tube.

At her 18-week follow-up from the rehab center, calcium remained normal and BP well-controlled. However, she continued to have residual deconditioning and could only follow two-step commands. No signs or symptoms of any malignancy had emerged, thus making paraneoplastic neurological syndrome much less likely and PRES with residual neurological impairment the most likely explanation.

## Discussion

PRES syndrome is both a clinical and radiological diagnosis with neurological changes coupled with MRI FLAIR hyperintensities in the parietal, occipital, and frontal bilateral areas [1-5]. This condition has been associated with uncontrolled hypertension, endothelial injury, and side effects from certain drugs, i.e., chemotherapy [5-7]. The theory behind the pathogenesis involves uncontrolled hypertension, which can cause elevated intracerebral pressure impairing autoregulation thus leading to extravasation of fluid from hyperperfusion, causing vasogenic edema. While, on the other hand, infections and autoimmune diseases can cause the release of cytokines and vasoactive substances like nitric oxide and thromboxane A2 causing vasoconstriction and endothelial injury. Similarly, cytotoxic injury from certain chemotherapy and immunosuppressant drugs causes injury to the endothelium destroying the blood brain barrier.

Thus far, as of April 30, 2022, there have not been any reports of COVID vaccine-related PRES syndrome through a narrative review of the PubMed, EMBASE, Google Scholar, and vaccine adverse event reporting system (VAERS) databases, in addition to UpToDate and DynaMed sources. The recent population-based cohort and self-controlled case series from the United Kingdom and Spain investigated 8.3 million cases of post-COVID vaccine neurological side effects [14]. The vaccines included in this recent analysis were mRNA vaccines: Pfizer and Moderna; vector vaccines: Jansen and Jansen and AstraZeneca; and one adjuvant recombinant spike protein nanoparticle vaccine: Novavax. The side effects post effects included Guillain-Barre syndrome, encephalomyelitis, Bell’s palsy, and transverse myelitis. However, there have not been any reported cases of PRES in this study but there have been cases post-COVID infection. As our case report met the clinico-radiological features of PRES without other identifiable causes or associations other than hypertension, we think it prudent to report the temporal relationship between the mRNA Moderna COVID vaccine booster in our patient. We cannot prove this beyond any reasonable doubt. 

The Centers for Disease Control and Prevention (CDC) offers providers a platform to report vaccine-induced side effects [15]. We did report this case as a possible VAER with the confounder of hypertension. We encourage consideration of reporting any neurological manifestations similar to our patient that occur post-vaccination to VAERS. Any neurological manifestations like altered mentation, uncontrolled hypertension, and seizure-like activity raise suspicion of PRES and should be followed up with CT and MRI imaging and should be reported.

As we mentioned above, reported cases of PRES do not always exhibit complete reversible neurological recovery. Whether timely management of cerebral perfusion pressure prevents permanent neurological damage, as seen in our patient who did not fully recover, remains to be determined.

## Conclusions

We emphasize the importance of timely diagnosis with MRI and preventing delays in management with adequate anti-hypertensives to potentially prevent irreversible neurological damage from PRES. We also encourage reporting similar neurological findings to VAERS, as we are unable to exclude a reaction to the mRNA vaccine booster.

## References

[1] Sudulagunta SR, Sodalagunta MB, Kumbhat M, Settikere Nataraju A (2017). Posterior reversible encephalopathy syndrome (PRES). Oxf Med Case Reports.

[2] Hobson EV, Craven I, Blank SC (2012). Posterior reversible encephalopathy syndrome: a truly treatable neurologic illness. Perit Dial Int.

[3] Parasher A, Jhamb R (2020). Posterior reversible encephalopathy syndrome (PRES): presentation, diagnosis and treatment. Postgrad Med J.

[4] Gewirtz AN, Gao V, Parauda SC, Robbins MS (2021). Posterior reversible encephalopathy syndrome. Curr Pain Headache Rep.

[5] Cherniawsky H, Merchant N, Sawyer M, Ho M (2017). A case report of posterior reversible encephalopathy syndrome in a patient receiving gemcitabine and cisplatin. Medicine (Baltimore).

[6] Foulser PF, Senthivel N, Downey K, Hart PE, McGrath SE (2022). Posterior reversible encephalopathy syndrome associated with use of Atezolizumab for the treatment of relapsed triple negative breast cancer. Cancer Treat Res Commun.

[7] Sommers KR, Skiles J, Leland B, Rowan CM (2022). Posterior reversible encephalopathy syndrome: Incidence and clinical characteristics in children with cancer. J Pediatr Hematol Oncol.

[8] Ammar Tarabichi, Faisal Ibrahim, Ahmed Abbas (2021). COVID-19 associated posterior reversible encephalopathy syndrome. Neurology Apr.

[9] Llansó L, Urra X (2020). posterior reversible encephalopathy syndrome in COVID-19 disease: a case-report. SN Compr Clin Med.

[10] Elhassan M, Saidahmed O, Adebayo A, Archibald N (2021). Persistent cortical blindness following posterior reversible encephalopathy syndrome (PRES) as a complication of COVID-19 pneumonia. Cureus.

[11] Motolese F, Ferrante M, Rossi M (2021). Posterior reversible encephalopathy syndrome and brain haemorrhage as COVID-19 complication: a review of the available literature. J Neurol.

[12] Gnanajothy R, Visserman LF, Sena KN (2017). Acute disseminated encephalomyelitis following meningococcal vaccination: case report and review of the literature. Conn Med.

[13] Sheta MA, Paladugu M, Mendelson J, Holland NR (2011). When should nitroglycerine be avoided in hypertensive encephalopathy?. Hypertension.

[14] Li X, Raventós B, Roel E (2022). Association between covid-19 vaccination, SARS-CoV-2 infection, and risk of immune mediated neurological events: population based cohort and self-controlled case series analysis. BMJ.

[15] (2021). COVID-19 vaccine reporting systems. https://www.cdc.gov/coronavirus/2019-ncov/vaccines/reporting-systems.html.

